# Predator hunting modes induce varied selection pressures on prey personality

**DOI:** 10.1093/beheco/arag097

**Published:** 2026-08-25

**Authors:** Rebecca K McKee, Storm Miller, Carl N Keiser, Marin P Chester, Kristen M Hart, Robert A McCleery

**Affiliations:** Department of Biology, Mercer University, Macon, GA 31207, United States; Department of Wildlife Ecology and Conservation, University of Florida, 110 Newins-Ziegler Hall, Gainesville, FL 32611, United States; Department of Wildlife Ecology and Conservation, University of Florida, 110 Newins-Ziegler Hall, Gainesville, FL 32611, United States; Department of Biology, University of Florida, 876 Newell Drive, Gainesville, FL 32611, United States; Department of Wildlife Ecology and Conservation, University of Florida, 110 Newins-Ziegler Hall, Gainesville, FL 32611, United States; Wetland and Aquatic Research Center, U.S. Geological Survey, 3321 College Avenue, Davie, FL 33314, United States; Department of Wildlife Ecology and Conservation, University of Florida, 110 Newins-Ziegler Hall, Gainesville, FL 32611, United States

**Keywords:** activity, behavior type, boldness, fitness, docility, prey behavior, temperament

## Abstract

Consistent individual differences in animal behavior—ie, personalities—have been documented in many taxa to have important fitness consequences in the context of predator-prey interactions. Multiple mechanisms have been suggested to maintain variation in prey personality traits, including life-history tradeoffs and divergent selection pressures from predators with different hunting strategies. However, empirical evidence for these hypotheses has been limited by a lack of data originating from complex multipredator systems in situ. To address this gap and evaluate these 2 mechanisms, we collected hispid cotton rats (*Sigmodon hispidus*) in the Florida Everglades and quantified 3 behavioral traits in the field. After releasing rats at their point of capture, we monitored their survival in their natural habitat where they faced threats from a diversity of ambush (snakes) and active predators (mammals; birds). Using Cox proportional hazards regression models and competing risks models, we assessed the effect of boldness, activity, and docility on rodent predation risk. Contrary to conventional predictions that bolder and more active individuals would face higher predation risk due to life-history tradeoffs, we found that the impact of these traits on prey survival varied with the predators' hunting modes: shyer, more active individuals were more likely to be killed by active predators, whereas less active rats appeared more likely to be killed by ambush predators. Our study supports the hypothesis that a diversity of predator hunting strategies may help maintain trait variation in prey populations.

## Teaser

We found that cotton rats, in the Florida Everglades, which showed different behavioral traits also experienced differing predation risk: shy, active rats were more likely to be killed by active predators (e.g., mammals, birds), but less active rats were more at risk for ambush predators (snakes). These findings suggest different predators may help maintain variation in prey behavior.

## Introduction

Consistent intraspecific differences in animal behavior, known as personalities, have been identified in a wide range of taxa (Dall et al. 2004; Bell et al. 2009; Brehm and Mortelliti 2018). Differences in personality traits such as activity level, exploration, and aggressiveness can influence foraging strategies (Patrick et al. 2017), habitat utilization (Brehm and Mortelliti 2021), and predator evasion tactics (Goodchild et al. 2020), ultimately affecting fitness (Dingemanse et al. 2004; Smith and Blumstein 2008). Like other characteristics, personality traits are subject to selection pressure (Dingemanse and Réale 2005). Accordingly, numerous hypotheses including life-history tradeoffs (Wolf et al. 2007; Réale et al. 2010; Brehm and Mortelliti 2024) and heterogeneity in selection pressures (Dochtermann et al. 2012), have been used to explain why populations maintain personality traits, despite apparent fitness costs (Sih et al. 2004; Réale and Dingemanse 2012).

Life-history tradeoffs occur when a trait is beneficial in one aspect of fitness but detrimental to another (Stearns 1989). It has been widely hypothesized that “bolder” or more risk-tolerant individuals will acquire more resources and experience faster growth rates but at the cost of lower survival due to their risky behavior (Réale et al. 2010). Conversely, “shyer” or more risk-averse individuals are predicted to survive longer and reproduce over longer time periods, resulting in similar relative fitness across personality types (Réale et al. 2010). The link between personality and survival is central to the Life-History Tradeoff Hypothesis, but empirical evidence supporting this hypothesis is mixed (Smith and Blumstein 2008; Carter et al. 2010; Hulthén et al. 2017; Roe et al. 2023). For example, a meta-analysis concluded that bolder individuals actually survived at higher rates than shyer individuals in wild populations (Moiron et al. 2020).

Another explanation for the persistence of personality traits is the presence of diverse or heterogeneous selection pressures (Dochtermann et al. 2012). Heterogeneity in selection pressure can result from differences in the density of conspecifics, competitors, and predators or variation in the availability of food and shelter (Nicolaus et al. 2016; Brehm and Mortelliti 2024). For example, selective pressures on bighorn sheep (*Ovis canadensis*) personality traits were only apparent in years of high cougar (*Puma concolor*) predation (Réale and Festa-Bianchet 2003). Predation specifically is a ubiquitous agent of natural selection (Johnson and Belk 2020) that has substantial influence on prey personality traits (Yli-Renko et al. 2018). Because prey species rarely experience predation risk from a single predator (Sih et al. 1998), predator community composition could also be a factor creating heterogeneity in predation pressure, and consequently, in trait selection. When selection pressure diverges between different predators, traits beneficial for evading 1 predator can inadvertently facilitate predation by another (Charnov et al. 1976; Embar et al. 2014). Consequently, patterns of predator-specific predation risk in multipredator systems could explain the lack of an empirical link between prey personality traits and survival outcomes. We refer to the maintenance of variation in prey traits due to varied selection pressures as the Predator Heterogeneity Hypothesis.

Predatory species in multipredator systems are likely to vary in many aspects of their physiology, ecology, and behavior (Schmitz 2017). Predator hunting mode (ie, sit-and-wait/ambush vs. actively hunting predators) is one particularly important characteristic impacting predator-prey interactions (Preisser et al. 2007; Miller et al. 2014; Kelleher et al. 2021; Sommer et al. 2023; Orrick et al. 2024) and may influence whether a given predator will consume more active or sedentary prey species (Huey and Pianka 1981). Specifically, the Locomotor Crossover Hypothesis predicts that more active predators will consume more sedentary prey, while ambush predators will consume more active prey (Huey and Pianka 1981). Although originally formulated to explain interspecific differences in predator-prey interactions, there is increasing interest in understanding whether intraspecific differences in predator hunting mode may similarly generate divergent selective pressures on prey personality types (Chang et al. 2017; Matsumura and Miyatake 2022). In addition to activity, predator hunting mode could influence selection of other prey personality traits, including boldness and docility (Belgrad and Griffen 2016; Blake et al. 2018). However, because many prior experiments assessing these patterns have been conducted in mesocosms or other artificial environments (Blake and Gabor 2014; Belgrad and Griffen 2016), evidence supporting the Predator Heterogeneity Hypothesis in natural systems where multiple predation risks occur simultaneously remains limited.

To assess evidence for the Life-History Tradeoffs Hypothesis and the Predator Heterogeneity Hypothesis in explaining the persistence of behavioral trait diversity in prey populations, we evaluated the behavior of a common rodent species and examined its survival in its natural environment, where it encountered threats from both actively hunting and ambushing predators. Our first objective was to test the prediction from the Life-History Tradeoff Hypothesis that individuals characterized as bolder, more active, or less docile would have higher predation risk. Our second objective was to test the prediction from the Predator Heterogeneity Hypothesis that different predator hunting modes exert divergent selection patterns on prey personality traits. Based on the predictions from the Locomotor Crossover Hypothesis, we expected ambush predators to remove more active prey and active predators to remove more sedentary prey.

## Materials and methods

### Experimental overview

We captured hispid cotton rats (*Sigmodon hispidus*) in the Greater Everglades (Florida, USA; hereafter Everglades) from April to August 2023. Following capture, we performed 3 behavioral tests to quantify their personality traits (McKee et al. 2026). To determine whether cotton rat personality traits influenced their predation risk, we tagged rats with VHF radiocollars equipped with mortality sensors. We monitored the signal of collared cotton rats every 24 to 48 h until the rat was consumed by a predator, the rat's collar failed, or the study concluded. We used survival analysis to assess the influence of behavioral traits on predation risk.

### Study site

Extending from Lake Okeechobee to the tip of peninsular Florida, the Everglades is an extensive wetland mosaic featuring wet prairies, sawgrass marshes, pine rocklands, and hardwood hammocks (Gunderson 1994). The Everglades is characterized by a subtropical climate with hot and humid summers (Richardson 2010), and includes Everglades National Park, the largest subtropical wilderness in the United States. Approximately 75% of the annual precipitation in the region falls between May and October (Abiy et al. 2019). Historically, the unique hydrology, mix of freshwater and saltwater biomes, and varied vegetation found within the Everglades has fostered a high degree of vertebrate biodiversity with 76 mammalian, 60 reptilian, and over 400 avian species recorded in Everglades National Park (Brown et al. 2006). To capture a range of predator communities, we selected 3 sites within the Everglades—Rocky Glades, C4, and Fran Reich. All 3 of these public lands are managed by the South Florida Water Management District. Despite differences in the predator communities, previous work has demonstrated that cotton rats experience similar rates or survival and predation risk across these sites, regardless of cotton rat sex (McCampbell et al. 2023).

### Study species

The hispid cotton rat is a widespread rodent that occurs in grass-dominated habitats throughout much of the southeastern and south-central United States (Cameron and Spencer 1981). Cotton rats are common prey for many mammalian, avian, and reptilian predators (Jones and Smith 1979; Barrett et al. 2001; McCampbell et al. 2023). Although they can live up to 23 mo in captivity, cotton rats usually live <6 mo in the wild as a result of predation (Curlee and Cooper 2012).

Within the Everglades, cotton rats face a diverse community of predators. Avian predators, including red-shouldered hawks (*Buteo lineatus*), red-tailed hawks (*Buteo jamaicensis*), barn owls (*Tyto furcata*), great horned owls (*Bubo virginianus*), and barred owls (*Strix varia*), are likely responsible for the majority of cotton rat predation events (McCampbell et al. 2023). Cotton rats also face a diverse community of snake predators, such as eastern diamondback rattlesnakes (*Crotalus adamanteus*), cottonmouths (*Agkistrodon conanti*), and rat snakes (*Pantherophis* spp.). Along with native snakes, cotton rats also are common prey of juvenile Burmese pythons (*Python bivittatus*), a constrictor native to southeast Asia that was introduced into Everglades National Park in the 1980s and 1990s (Guzy et al. 2023; Romagosa et al. 2023). In addition to depredating cotton rats, python establishment in the Everglades has coincided with reductions in populations of native mammalian predators (Dorcas et al. 2012; Buckman et al. 2024), including bobcats (*Lynx rufus*), gray foxes (*Urocyon cinereoargenteus*), and red foxes (*Vulpes vulpes*). However, mammalian predators are still detected in regions where pythons are less numerous and in areas proximate to urban areas that may buffer the impacts of pythons (Reichert et al. 2017; Taillie et al. 2021). Although historically absent from southern Florida, coyotes (*Canis latrans*) are now common in much of the Everglades and may not experience the same python-associated declines as other mammalian predators (Reichert et al. 2017).

In addition to taxonomic diversity, cotton rat predators present across the Everglades use a wide range of hunting modes that fall along the spectrum between ambush and active search tactics. For the purposes of this study, we categorized predators as using primarily ambush or primarily active hunting modes. Due to metabolic and morphological constraints that limit their ability to pursue endothermic prey, snakes were classified as relying more heavily on ambush hunting tactics. In contrast, mammalian predators and raptors were placed at the more active end of the hunting mode spectrum (Dell et al. 2014; Hanscom et al. 2023). While we acknowledge that snakes, mammals and bird may rely on multiple tactics to capture prey (Glaudas et al. 2019), there is a clear separation in broad hunting strategies of endothermic and ectothermic predators (Dell et al. 2014) of cotton rats.

### Rodent capture and processing

To optimize capture of cotton rats, we placed Sherman traps in areas dominated by grassy vegetation (Kincaid and Cameron 1985) and baited them with bird seed. During periods of heavy rain fall, we placed floats underneath traps to prevent them from becoming submerged (McCleery et al. 2022). Traps were set roughly 2 to 3 h prior to sundown and were checked within 2 to 3 h of sunrise to minimize heat stress on the rodents. Upon capture, we carried the cotton rats in their trap to the centrally located behavioral arena.

### Behavioral assays

**Figure 1:**
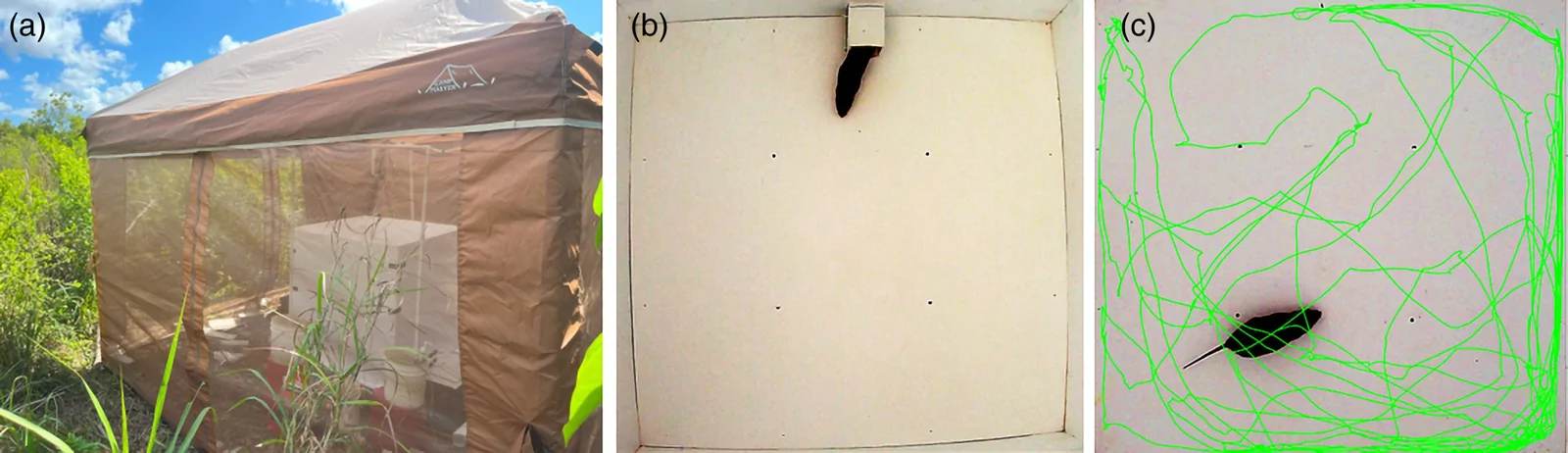
Experimental setup for hispid cotton rat (*Sigmodon hispidus*) personality tests conducted at 3 sites in the Everglades (Florida, USA). a) Behavioral processing tent that provided uniform shade and housed the behavioral arena used for emergence tests (ET), open field tests (OFT), and handling bag tests. b) Rat on verge of fully exiting tunnel in ET used to quantify shyness. c) Example movement pattern of a cotton rat during a 5-min OFT trial to quantity activity.

The behavioral arena was an opaque square box (90 × 90 × 90 cm), a design commonly used with larger rodents (Boon et al. 2007; Brehm and Mortelliti 2018). To standardize canopy cover, we placed the arena under the shelter of a tent (3.05 × 3.05 m) and covered tent walls with cloth shades to reduce direct sunlight (Fig. 1a). We positioned an EK7000 action camera (AKASO; Frederick, MD) above the arena on a PVC pole mount to video record trials (4k/30 fps). We cleaned the arena between individuals by wiping the arena floor and lower walls with a 7% bleach solution to reduce the influence of residual conspecific odors and minimize the spread of disease. To quantify personality traits of cotton rats, we used 3 behavior assays commonly employed in a variety of rodent species (Brehm and Mortelliti 2018).

Emergence tests—occasionally called modified open field or latency tests—have been widely used in personality assays as a measure of boldness (Carter et al. 2013; Brehm and Mortelliti 2018). Because enclosed spaces like traps feel safe, increased latency to emerge or failure to emerge is considered an indication of risk-aversion or shyness, whereas quick emergence indicates risk-tolerance or boldness (Carter et al. 2013). Prior to the start of the test, we equipped the Sherman trap with an opaque tunnel. We initiated the emergence test (ET) by removing the sliding door from the tunnel, allowing rats to enter the arena at will (Fig. 1b). After this barrier was removed, we left the processing tent to avoid observer influence on rat behavior. Emergence time was monitored using a Bluetooth connection to the arena camera. If a rat emerged from the tunnel to the extent that all 4 feet were planted in the arena, we considered the rat to have fully emerged. Following the rat's emergence, we lowered a Sherman trap into the arena to recapture the rat prior to the next test. Recapturing rats that emerged allowed us to begin the subsequent tests in the same manner for all rats, regardless of their emergence status (Brehm et al. 2019). If a rat did not fully emerge within 10 min, we marked that the rat failed to fully emerge and concluded the test. We reviewed video footage of the ET to record the exact time (in seconds) that rats emerged into the arena and used this measure to quantify shyness. If the rat did not emerge, we recorded its emergence score as the full amount of time as the test (600 s).

Open field tests (OFT) were used to capture the overall activity level and exploratory behavior of individuals (Gould et al. 2009; Brehm et al. 2020). By placing individuals in a larger arena, ie, an open field, these tests quantify activity with metrics like total distance moved, average speed of movement, and time spent moving (Gould et al. 2009). After the ET was complete, we transferred the rat from the Sherman trap into the center of the same arena to begin the open field test. We recorded the rat for 8 min, excluding the initial 30 s of footage following its introduction into the arena to mitigate any potential impact of the researcher's departure from the tent on the rodent's behavior (ie, this was treated as an acclimation period). Because some trials were cut short due to camera difficulties, we analyzed only the first 5 min of video from each rat for consistency. We used the open-source software ToxTrac to track cotton rat movements in the arena (Rodriguez et al. 2018). Using ToxTrac (Rodriguez et al. 2018), we recorded each rat's total distance moved, average speed, average moving speed (ie, the speed of the rat when it was moving), time spent inactive, and exploration for the 5-min clip (Fig. 1c).

The handling bag test (HBT) assesses docility in rodents by measuring their response to human handling, which serves as a proxy for a predator encounter (Martin and Réale 2008; Brehm and Mortelliti 2018; Brehm et al. 2020). A higher proportion of time spent frozen during handling indicates greater docility. After the OFT was complete, we recaptured the rat by placing a Sherman trap in the arena. We then transferred the rat to a semi-transparent, zippered mesh handling bag to assess the rat's docility. To ensure rats were held at the same height and in the same position in each trial, we fit the loop at the top of the handling bag onto a PVC pole. We then placed the PVC pole across the top of the OFT arena so that the top of the handling bag was 90 cm above the floor and in the center of the arena. We observed the rat in real time, recording the number of seconds it moved during a 1-min period with a stopwatch.

One core tenant of animal personality is consistency or repeatability in behavioral traits across time (Réale et al. 2007; Bell et al. 2009). To show that cotton rats behaved consistently, we placed an ear tag with a unique ID on each rat and repeated our behavioral assay in cotton rats at our 3 study sites that were either never collared or were not yet collared. We had an interval of at least 5 d between tests for rats that underwent repeated testing. In order to reduce variation across trials, the 3 tests were always conducted in the same order. In total, we conducted 257 trials on 165 individuals with 64 rats tested on at least 2 occasions for the ET; 252 trials on 164 individuals with 62 rats tested on at least 2 occasions for the OFT; and 252 trials on 165 individuals with 61 rats tested on at least 2 occasions (Table 1; McKee et al. 2026). Slight deviations in sample size between the 3 behavioral tests were due to camera malfunctions or the accidental release of individuals between tests.

**Table 1:** Results of repeatability analysis with estimated intraclass correlation coefficients (R), bootstrapped 95% confidence intervals, likelihood ratio test deviances (D), and corresponding *P*-value for cotton rat behavioral traits in uncollared cotton rats (*Sigmodon hispidus*).

Trait	Test	Metric	Observations [individuals]	*R* [95% CI]	*D*	*P*-value
Shyness	ET	Seconds to emerge	257 [165]	0.27 [0.07 to 0.45]	11.4	<0.001
Activity	OFT	Distance moved (cm)	252 [164]	0.26 [0.05 to 0.44]	6.29	0.006
Activity	OFT	Speed (mm)	252 [164]	0.28 [0.07 to 0.47]	7.71	0.003
Activity	OFT	Mobile speed (mm)	252 [164]	0.30 [0.09 to 0.49]	8.93	<0.001
Activity	OFT	Seconds frozen	252 [164]	0.31 [0.11 to 0.50]	11.4	<0.001
Exploration^a^	OFT	Number of squares	252 [164]	0.51 [0.32 to 0.66]	19.9	<0.001
Docility^a^	HBT	Seconds frozen when handled	251 [165]	0.50 [0.32 to 0.65]	17.9	<0.001

Cotton rats were subjected to 3 behavioral tests, an ET, an Open Field Test (OFT), and a HBT.

^a^Modeled as an inverse to better fit distribution.

### Radio telemetry and mortality categorizations

Immediately after behavioral tests were complete, we weighed rodents to the nearest gram using a spring scale and placed a metal ear tag with a unique number to identify individuals upon future captures. We placed a radio collar (model number SOM-2190; Wildlife Materials, Murphysboro, IL) that included a mortality sensor on adult rats >130 g. Mortality sensors were activated if no movement was detected over an 8-h period. We monitored each rat's signal every 24 to 48 h to determine if the rat was alive. If a mortality signal was detected, we tracked the signal to determine the likely cause of death. We attributed deaths to snake predation if the transmitter was located inside a snake, was regurgitated, or was located in snake scat (McCleery et al. 2015; McCampbell et al. 2023). If we observed teeth marks, mammalian scat or tracks at the kill site, we attributed the mortality to a mammalian predator (Conner et al. 2011). We considered feathers or avian excretions at the kill site, beak marks on the collar, and partially picked apart carcasses to indicate avian predation (Conner et al. 2011). In addition to checking mortality signals, we also tracked each rat at least once each week to try to visually confirm each rat was alive. Rats were collared between April 14 and 8 August 2023. Rats were monitored from their time of collaring until 1 October 2023. In total, we recorded behavior data of and fitted radio-collars on 98 adult rats, including 37 at Rocky Glades, 31 at C4, and 30 at Fran Reich (McKee et al. 2026). Of these, 62 were male and 36 were female. Our work was conducted in accordance with the Institutional Animal Care and Use Committee (permit #202200000246).

### Statistical analyses

#### Repeatability and relationships between personality metrics

We conducted repeatability analyses by calculating intraclass correlation coefficients (R) on personality metrics before using them in our analyses (Hayes and Jenkins 1997). Values of R fall between 0 and 1, with 0 indicating no repeatability and 1 indicating complete repeatability (ie, behaviors are highly repeatable across time and individuals are consistently different from each other). We used the package rptR to estimate R values, bootstrap 95% confidence intervals, and conduct likelihood ratio tests between the model with and without individual ID (Stoffel et al. 2017). We considered metrics with an R estimate above 0.2 to indicate repeatability (Kuo et al. 2023), provided the bootstrapped 95% confidence interval also did not include 0 (Table 1). In cases where we assessed the same adult rat (mass >110) on multiple occasions prior to collaring, we used the average of its scores on behavioral tests to assess correlations between metrics and in future survival analyses.

We then assessed the correlation between metrics using by calculating Spearman's rho rank correlation coefficients in the package Hmisc (Harrell 2024). We categorized correlation coefficients of 0.00 to 0.19 as very weak, 0.20 to 0.39 as weak, 0.40 to 0.59 as moderate, 0.60 to 0.79 as strong and 0.80 to 1.00 as very strong evidence of correlation between test metrics (Younes et al. 2021). In personality studies, many studies rely on Principle Components Analysis (PCA) to reduce the number of variables and avoid including highly correlated covariates in models (Budaev 2010). This approach results in a composite measure or measures that reflect many aspects of an individual's personality in orthogonal, independent axes. However, biological interpretation of composite metrics is often less straightforward (Brehm and Mortelliti 2018). Because of our interest in the effect of each trait on survival, we elected to include only a single metric from each behavioral test to reduce correlated covariates in our model while also facilitating biological interpretability. An alternative analysis using a PCA of OFT metrics yielded similar results (Supplement S1).

#### Survival analysis

We used nonparametric Kaplan–Meier survival curves to estimate overall survival of cotton rats (Kaplan and Meier 1958). To determine whether cotton rat personality traits influenced their survival, we used a combination of survival analysis techniques. To assess support for the Life-History Tradeoff Hypothesis, we used Cox proportional hazards regression models (Cox 1972). This semi-parametric model assesses the effect of covariates on survival and is a common analytical technique for wildlife telemetry studies (Riggs and Pollock 1992). These models incorporate time to event data and are beneficial because they allow the inclusion of both data where the event of interest occurs and cases where the event of interest is not observed (ie, censored observations). We used a Cox model to determine if shyness (emergence time in the ET), activity (distance moved in the OFT), and docility (seconds frozen when handled in the HBT) impacted a rat's survival probability. In this model, days between collaring and death was the response variable and all 3 personality traits were included as covariates. We treated death attributable to any predator as an event. We censored any observations when the rat was alive at the end of the study, there was evidence of collar failure, or the death was due to old age or nonpredatory causes. We also censored rats whose deaths could be attributed to capture or collaring (eg, rat died within 72 h of collaring). We implemented both Kaplan–Meier and Cox models in the package survival (Therneau 2023) in R (R Core Team 2023). We also used this package to test whether the assumption of proportional hazards was met (Grambsch and Therneau 1994; Supplement S2). We considered a *P*-value ≤0.1 but >0.05 to be weak evidence, a *P*-value between 0.05 and 0.01 to be moderate evidence, and a *P*-value ≤ 0.01 to be strong evidence of an effect of a personality trait on survival (Muff et al. 2022; Peignier et al. 2023).

To assess support for the Predator Heterogeneity Hypothesis and determine whether the effect of cotton rat personality traits varied by predator hunting modes, we used cause-specific Cox regressions (CSC; Cox 1972; Prentice et al. 1978) and Fine–Gray proportional hazards regression (FGR) models for competing risks (Fine and Gray 1999). Both CSC and FGR are extensions of Cox proportional hazards regressions models, allowing for the analysis of scenarios with competing risks (Austin and Fine 2017; Zhang et al. 2018). Cause-specific hazards models assess the effect of covariates on hazard function for a specific risk, such that each risk of interest is modeled separately (Zhang et al. 2018). In contrast, FGR models estimate the cumulative incidence function and assess the effects of covariates on the sub-distribution hazard function for each competing risk (Austin and Fine 2017). Although these 2 approaches are similar, they convey slightly different pieces of information (Latouche et al. 2013). Accordingly, we report results from both methods, as is commonly recommended (Latouche et al. 2013; Zhang et al. 2018). We used these models to compare the effects of personality traits on mortality from predators with active hunting modes (mammals and birds) versus ambush predators (snakes). We categorized deaths by mammalian/avian predators as cause 1, snake predators as cause 2, and censored any records where either a death did not occur, or it could not be attributed to either of these categories. We used the packages cmprsk and risk regression to implement CSC and FGR models (Gerds and Kattan 2021; Gray 2022; Gerds et al. 2023).

## Results

### Behavioral repeatability and relationships between personality metrics

We observed evidence of personality traits in cotton rats, with repeatable metrics generated from all 3 behavioral tests (Table 1). We observed strong correlations between metrics arising from the OFT, but not between metrics from different behavioral tests (Table 2). Accordingly, we included both seconds to emerge (a measure of shyness) and seconds inactive during the HBT (a measure of docility) as covariates in survival models. In order to minimize correlation between OFT variables and facilitate interpretation of our results, we incorporated distance moved during the OFT as our measure of activity (Strijker et al. 2023) in general predation and competing risk models.

**Table 2:** Spearman's rho correlation coefficients between cotton rat personality metrics assessed through 3 behavioral tests, the ET to quantify shyness, Open-field Test (OFT) to quantify activity, and HBT to quantify docility in hispid cotton rats hispid cotton rats (*Sigmodon hispidus*) in the Everglades (Florida, USA).

Test	Metric	Emergence	Speed	Distance	Moving speed	Secs frozen	Exploration	Docility
ET	Seconds to emerge	—	−0.34	−0.35	−0.34	0.32	−0.37	0.25
OFT	Speed	−0.34	—	1.00	0.91	−0.86	0.80	−0.22
OFT	Distance	−0.35	1.00	—	0.90	−0.86	0.80	−0.22
OFT	Moving Speed	−0.34	0.91	0.90	—	−0.63	0.68	−0.23
OFT	Secs Frozen	0.32	−0.86	−0.86	−0.63	—	−0.80	0.18
OFT	Exploration	−0.37	0.80	0.80	0.68	−0.80	—	−0.26
HBT	Seconds frozen	0.25	−0.22	−0.22	−0.23	0.18	−0.26	—

In cases where a rat was assessed multiple times as an adult prior to collaring, we first computed an average score of each individual for each of the metrics assessed.

### General predation models

**Figure 2:**
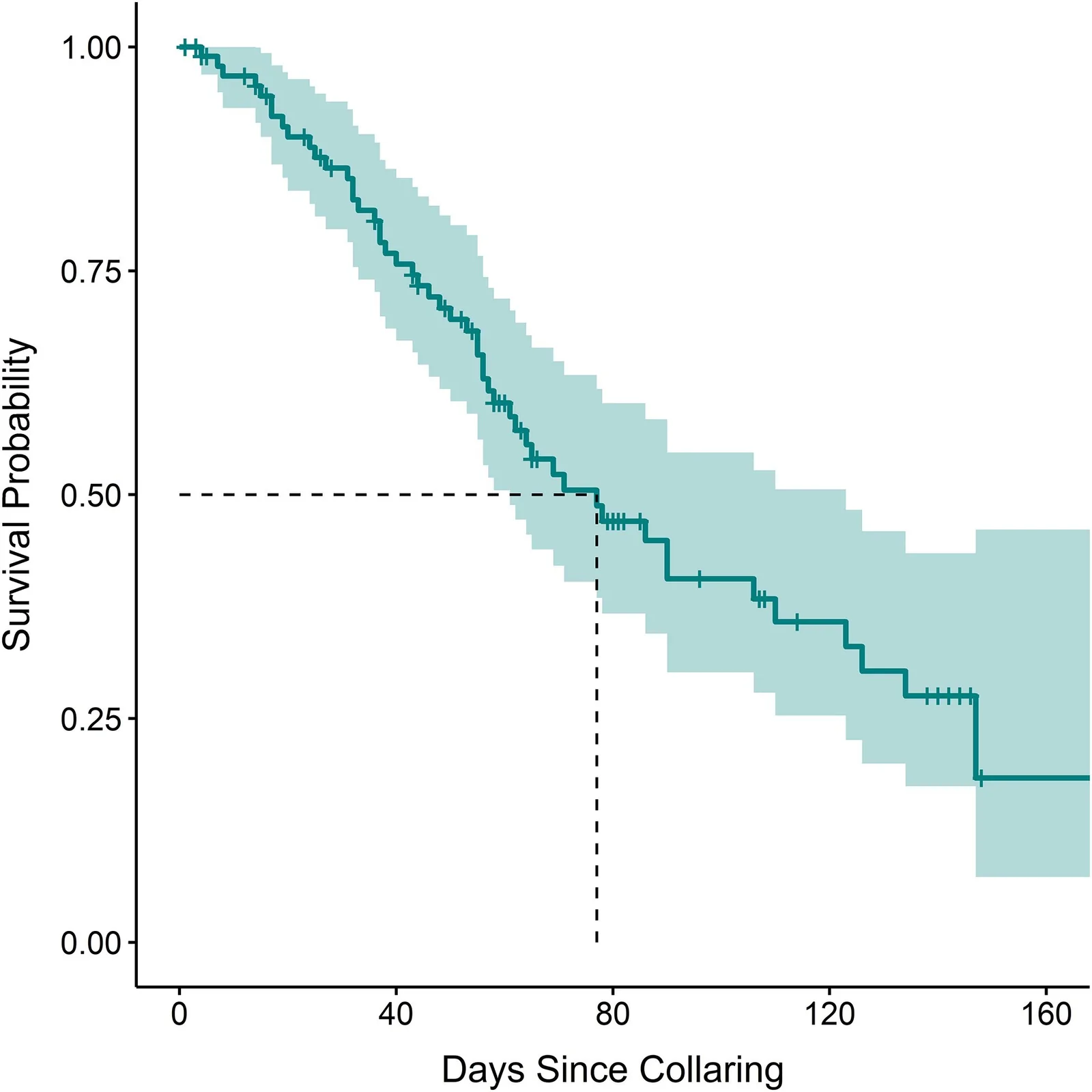
Kaplan–Meier survival curve for hispid cotton rat (*Sigmodon hispidus*) survival based on data collected in a radio-telemetry study. Model estimates are based on 50 predation events and 48 censored observations. Shaded area indicates 95% confidence interval of estimates. Dashed lines indicate median survival.

**Figure 3:**
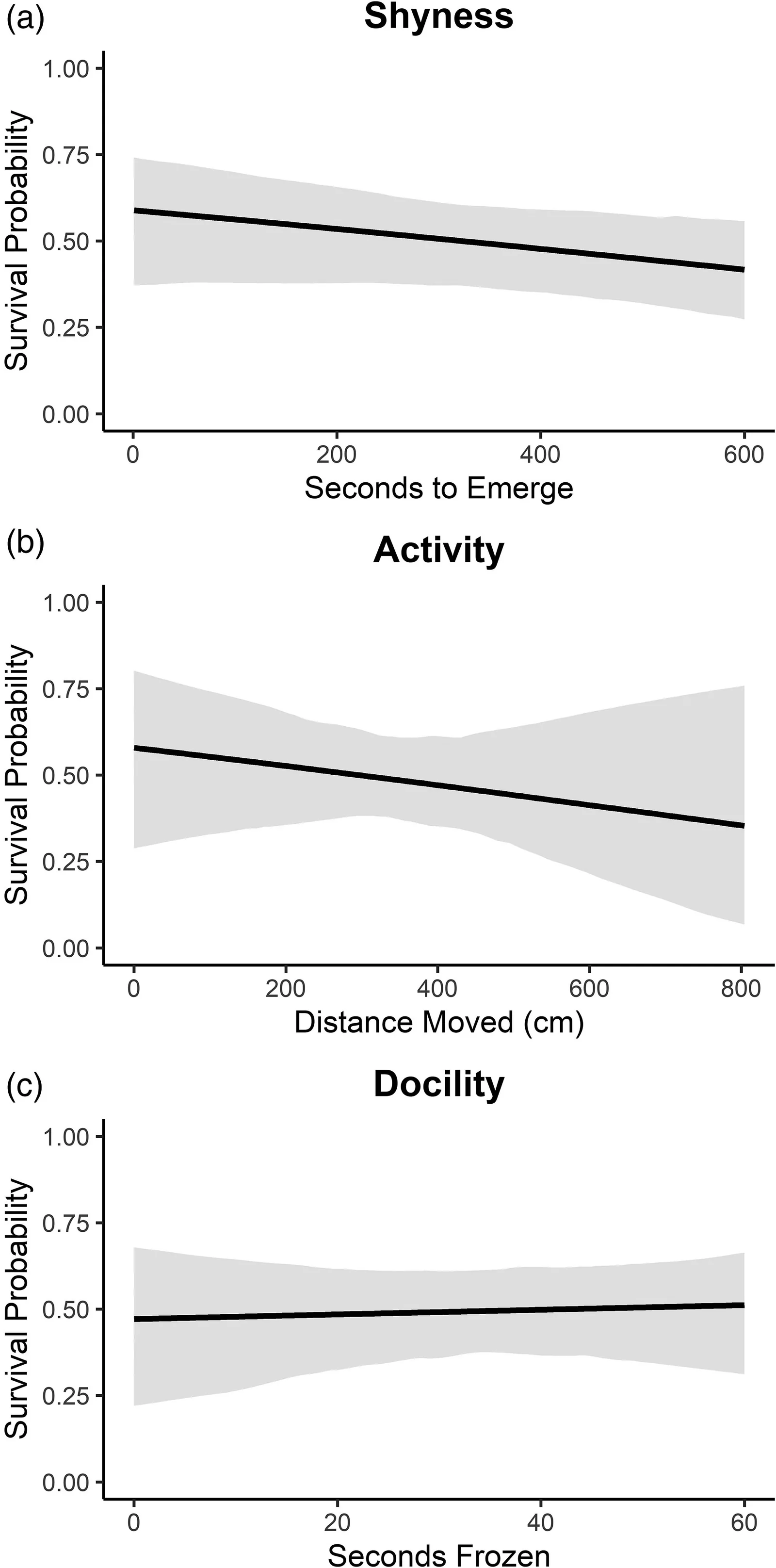
Cox proportional hazards regression models depicting the relationship between cotton rat (*Sigmodon hispidus*) personality traits: a) shyness, b) activity, and c) docility and survival probability. Relationships between each variable and survival are plotted at 77 d (median survival for cotton rats in the study). Gray shading indicates 95% confidence intervals.

We recorded a total of 50 deaths attributable to predation events (*n* = 27 mortalities from avian predators, *n* = 6 mortalities from mammals, *n* = 11 predation events from snakes, and *n* = 6 events from unknown predators). Kaplan–Meier estimates indicated a median survival of 77 d (95% CI: 61 to 123; Fig. 2). Contrary to the predictions of the Life-History Tradeoff Hypothesis, we found no evidence that personality traits influence cotton rat predation risk (Table 3; Fig. 3). Cox proportional hazards regressions revealed no significant relationship between shyness (*z* = 1.28; *P* = 0.20), activity (*z* = 0.81; *P* = 0.42), or docility (*z* = −0.23; *P* = 0.82) and survival (Table 3; Fig. 3).

**Table 3:** Cox proportional hazards regression model estimates for 3 personality metrics assessed in hispid cotton rats (*Sigmodon hispidus*) in the Everglades (Florida, USA).

Trait	Coefficient	Exp (coefficient)	SE	*Z*	*P*-value
Shyness	0.0008	1.0008	0.0007	1.301	0.20
Activity	0.0008	1.0008	0.0010	0.950	0.42
Docility	−0.0019	0.9980	0.0084	−0.233	0.82

Estimates were based on cotton rat radio-telemetry data with 50 events (deaths attributed to predation) and 48 censored observations.

### Cause-specific mortalities and competing risk models

**Figure 4:**
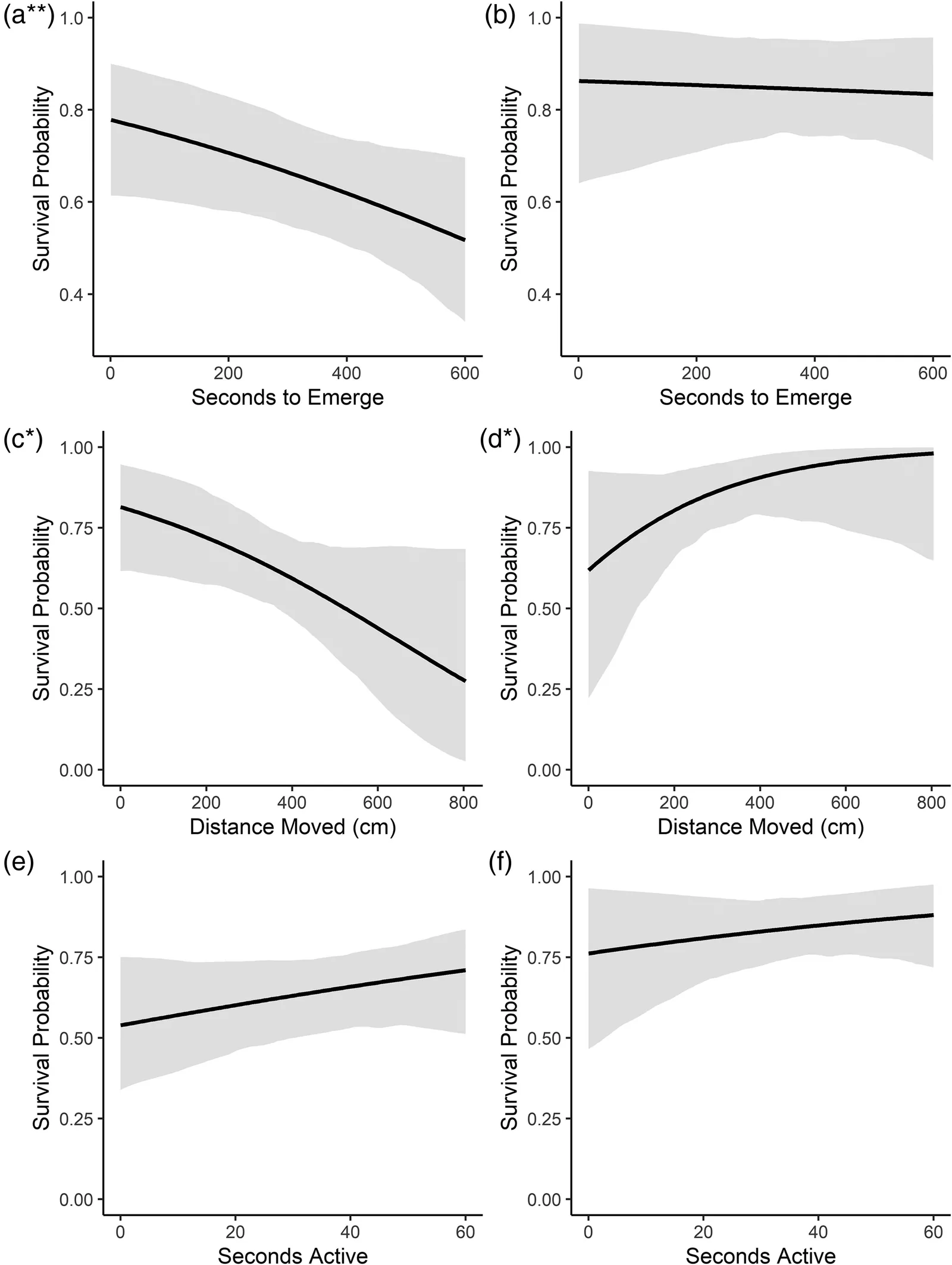
Relationships between personality metrics and survival based on cause-specific cox proportional hazards regression models for active predators (a, c, e) and ambush predators (b, d, f). Relationships between each variable and survival are plotted at 77 d (median survival for cotton rats in the study). Gray shading indicates 95% confidence intervals. **Indicates a *P*-value < 0.05. *Indicates a *P*-value ≤ 0.10.

We observed 33 deaths by the more active pursuit predators (birds/mammals) and 11 deaths attributable to ambush predators (snakes). We found evidence that the relationship between personality covariates and risk differed between active and ambush predators, indicating that predator hunting mode may be a source of heterogeneity in predation risk and consequently, selection pressure. Fine-gray models indicated that shyer individuals had higher risk in the context of pursuit predators (*z* = 2.01, *P* = 0.045; Table 4), but no such pattern existed with ambush predators (*z* = 0.17, *P* = 0.87; Table 4). Coefficients from CSC model for active predators indicated that a 100-s delay in emerging correlated with a hazard ratio of 1.185, or an 18.5% increase in the likelihood of predation (Fig. 4a). Consequently, a rat emerging mid-way through the test (300 s in) would have a ∼66% higher risk of dying from an active predator than a rat that emerged immediately.

**Table 4:** Fine-gray competing risk regression model estimates for hispid cotton rat (*Sigmodon hispidus*) deaths in the Everglades (Florida, USA) attributed to predators with active hunting modes (mammalian/avian predators) and sit-and-wait, ambush predators (reptiles).

Predator	Trait	Coefficient	Exp (coefficient)	SE	*Z*	*P*-value
Active	Shyness	0.0017	1.0017	0.0008	2.02	0.045**
	Activity	0.0030	1.0030	0.0013	2.26	0.024**
	Docility	−0.0107	0.9894	0.0089	−1.20	0.230
Ambush	Shyness	0.0002	1.0002	0.0013	0.167	0.870
	Activity	−0.0040	0.9960	0.0023	−1.74	0.083*
	Docility	−0.0139	0.9862	0.0186	−0.746	0.460

*Indicates *P*-value ≤ 0.10. **Indicates *P*-value ≤ 0.05.

In opposition to the predictions of the Locomotor Crossover Hypothesis, FGR models indicated that more active rats had a higher risk of death by active predators (*z* = 2.26, *P* = 0.02; Table 4). In our CSC model, moving 100 cm more in the OFT was linked to a 27% increase in the likelihood of being killed by an active predator (Fig. 4c). Intriguingly, we observed some evidence for the opposite trend with activity and survival in the context of ambush predators (*z* = −1.74, *P* = 0.08; Table 4; Fig. 4d; Table S5). In contrast with the CSC for active predators, a 100-cm increase in distance moved resulted in a hazard ratio of 0.67 in the context of ambush predators. We did not observe a pattern with docility and survival for predators of either hunting mode (Table 4; Fig. 4e, f).

## Discussion

Contradicting predictions from the Life-History Tradeoff Hypothesis, we found no evidence that bolder, more active, and less docile animals incurred higher risks of predation (Réale et al. 2010), likely due to heterogeneous pressures exerted by different predators. Consistent with the Predator Heterogeneity Hypothesis, we found that personality traits impacted a cotton rat's risk of predation differently in the context of active versus ambush predators (Table 4, Fig. 4). As one of the only field-based studies linking personality to predation outcomes in a complex multipredator system, our results add to a growing body of evidence that heterogeneity in selection pressures maintains prey personality traits (Dochtermann et al. 2012; Laskowski et al. 2022; Brehm and Mortelliti 2024). In addition to variation in predator density and predation rates that have long been linked to changes in selection pressure (Réale and Festa-Bianchet 2003; Bell and Sih 2007), we suggest that the composition of predators warrants additional consideration as an important mechanism for maintaining prey personality traits.

Our finding that predator hunting mode influenced the relationship between prey personality traits and survival in the field aligns with previous laboratory experiments (Belgrad and Griffen 2016; Blake et al. 2018). Although a growing number of studies have observed a relationship between prey boldness and predator modes (Belgrad and Griffen 2016; Blake et al. 2018), the direction of such relationship has not been consistent across studies. Using a fish model, Blake et al. (2018) found that ambush predators selectively removed shy prey, but the active predator removed both shy and bold prey evenly (Blake et al. 2018). This finding was partially consistent with a study on mud crabs, where shyer individuals were also selectively consumed by an ambush predator (Belgrad and Griffen 2016). In our study however, shyer, more risk-averse rats had a higher predation risk with active—but not ambush—predators. This pattern could indicate that the interactive effects between hunting mode and prey personality may be system or even species-specific, failing to conform to a universal pattern (eg, that active predators prefer bold prey, while ambush predators prefer shy prey). Should such a universal pattern exist, many factors, including challenges with accurately defining and quantifying boldness across a range of species (Carter et al. 2013), methodological differences between studies (Näslund et al. 2015), and/or the context (ie, laboratory, field) of the study (Moiron et al. 2020) could obscure overarching trends, complicating the link between prey personality traits and predator hunting mode.

We found that the relationship between prey activity level and predation risk also differed between predator hunting modes (Table 4). Prey that moved greater distances in OFT trials had lower survival in the context of active predators (Fig. 4c) but better survival in the context of ambush predators (Table 4; Fig. 4d). In the context of cotton rats specifically, it is possible that more mobile individuals were easier for active predators to detect and locate. For example, red-shouldered hawks, one of the most common predator species in this system (Marain 2020), have specialized search strategies adapted for detecting prey movement in relatively-open canopy systems (O’Rourke et al. 2010). If rats that moved further in the OFT also moved more frequently or over larger distances than less active rats, this could result in greater predation opportunities by birds and mammals. Conversely, because many snakes select ambush sites based on the chemosensory cues of prey (Clark 2004), rats that moved less may have generated more concentrated and reliable cues, making these individuals easier to locate and reliably target.

Our observation that more active rats were more likely to be consumed by active predators contradicts the Locomotor Crossover Hypothesis, which predicts a “crossover” between prey activity and predator hunting mode, such that more active predators remove more sedentary prey while ambush predators remove more active prey (Huey and Pianka 1981). Although this idea is intuitive and has been broadly accepted at the species level (Schmitz 2017), its applicability to intraspecific variation in prey activity remains uncertain (Chang et al. 2017; Matsumura and Miyatake 2022). In our study, we examined guild-level variation among predators and individual-level differences among prey and found little evidence to support the theory's predictions. One potential limitation is our binary classification of predators (as ambush or active hunters) based on broad taxonomic groupings. While this approach may seem course, these classifications are supported by thermal biology patterns in predator-prey pairings (Dell et al. 2014) and by trends observed in our data (Supplement S3). Future studies with larger sample sizes and more refined classifications of predator hunting strategies can help to more rigorously test the Locomotor Crossover Hypothesis and to clarify how predator traits interact with intraspecific variation in prey behavior.

Although we attribute the observed predation patterns to personality differences among individuals, we cannot rule out other factors that may independently or interactively influence an individual's predation risk. For example, intrinsic state-dependent variables such as age, parasite load, reproductive status, and body condition may influence individual behavior (Wolf and Weissing 2010; Sih et al. 2015) and vulnerability to predation (Hudson et al. 1992; Murray 2002; Karell et al. 2010). To minimize the influence of handling and capture on rodent predation risk, we did not attempt to recapture individuals following collaring. Accordingly, our study design did not permit assessment of an individual's state (eg, mass, reproductive status, apparent health) close to the time of mortality. Understanding how state-related traits interact with personality to shape predator-specific survival remains an important avenue for future work. However, existing evidence suggests that many rodent personality traits show little state dependence (Underhill et al. 2021) and a meta-analysis across a wider range of taxa found minimal evidence for the influence of individual states on personality (Niemelä and Dingemanse 2018).

Despite some compelling trends that support the notion that the effect of prey personality trait on survival varies across predators, we acknowledge that our results should be interpreted somewhat cautiously due to the low and unevenly distributed number of predation events observed (*n* = 33 for active predators, *n* = 11 for ambush predators). Generally, it is recommended to have at least 10 events per predictor variable for Cox models (Peduzzi et al. 1995; Vittinghoff and McCulloch 2007).Given our 3 focal personality traits, a minimum of roughly 30 events per predator group would have been recommended. If our observed predation rates are representative of this system, very large sample sizes of rodents (*n* = 300) may be required to achieve sufficient power to more precisely quantify the effects of personality traits on depredation by ambush predators. Nevertheless, the fact that we observed no significant effect of personality traits in a general predation model with a larger sample size, but some significant effects of personality in cause-specific models lends some confidence to our findings. Still, small sample sizes limited our ability to assess other influential factors—such as site, sex, and their interactions with personality—that might also shape predation risk. Although recent research in this system found no strong effects of sex or site on cotton rat survival (McCampbell et al. 2023), studies in other systems suggest these factors can interact with behavioral traits to influence predation risk (Yli-Renko et al. 2018; Brehm and Mortelliti 2024).

## Supplementary Material

arag097_Supplementary_Data

## Data Availability

Analyses reported in this article can be reproduced using the data published by the authors (McKee et al. 2026).
