# Supplementary material for: Predator hunting modes induce varied selection pressures on prey personality
Source: Behav Ecol. 2026 Aug 25;37(5):arag097. doi: 10.1093/beheco/arag097 (PMC13540721; doi:10.1093/beheco/arag097)
Supplement: Supplementary file 1 [file arag097_supplementary_data.pdf]

Supplementary Materials

Supplement 1: Principal Component Analysis (PCA) of Open Field Test metrics

Background

Based on Spearman's correlation coefficients calculated using the *Hmisc* package (Harrell 2024), metrics from the Open Field Test (OFT) were correlated (*rho > 0.5*). Accordingly, we conducted a Principal Component Analysis (PCA) of the five traits: distance moved, speed, mobile speed, time frozen, and exploration. To improve the normality of the data, we log-transformed time frozen and applied the square root of the inverse of exploration (100 minus the number of squares explored). We used the Anderson–Darling test for univariate normality on the transformed variables using the R package *MVN* (Korkmaz et al. 2014). Although the log transformation of time frozen improved normality, the transformed variable still showed evidence of deviating from normality (Table S1). Because normality is not required for PCA, and because we wished to include all repeatable metrics in our analysis, we retained the log-transformed time frozen variable in the PCA (Jolliffe 2002). After standardizing the metrics, we conducted a PCA and report the factor loadings in Table S2. The first principal component explained the vast majority of the variation (88%; Table S3).

Because the first principal component explained such a high proportion of the variance, we used it as the activity measure in Cox proportional hazards models (Table S4). We also used this measure in Fine-Gray competing risks models to determine whether predator selection patterns differed between ambush predators (snakes) and active predators (birds and mammals; Table S5).

Table S1. Anderson-Darling univariate normality tests conducted on five metrics generated from open field tests.

Variable	Statistic	P-value
Distance moved (cm)	0.53	0.17
Speed (mm)	0.55	0.15
Mobile speed (mm)	0.49	0.21
Log (Seconds frozen)	0.95	0.02
Sqrt (Inv.Exploration)	0.64	0.09

Table S2. Factor loadings from principal components analysis of open field test metrics in cotton rat behavioral assays.

	Comp.1	Comp.2	Comp.3	Comp.4	Comp.5
Speed (mm)	0.47	0.17	0.23	0.43	0.72
Distance moved (cm)	0.47	0.16	0.23	0.46	0.70
Mobile speed (mm)	0.43	0.65	-0.27	-0.57	---
Log (Seconds frozen)	-0.44	0.53	-0.51	0.52	---
Sqrt (Inv.Exploration)	-0.42	0.49	0.75	-0.12	---

Table S3. Importance of components in principal components analysis of cotton rat behavioral metrics collected in an open field test.

	Comp.1	Comp.2	Comp.3	Comp.4	Comp.5
Standard deviation	2.08	0.60	0.47	0.19	0.02
Proportion of variance	0.88	0.07	0.04	0.01	0.00
Cumulative proportion of variance	0.88	0.95	0.99	1.00	1.00

Table S4. Cox proportional hazards regression model estimates for three personality metrics, with activity represented by the first component of a principle coordinate analysis of five metrics recorded in a 5-min open field test. Estimates were based on cotton rat radio-telemetry data with 50 events (deaths attributed to predation) and 48 censored observations.

Trait	Coefficient	Exp (coefficient)	SE	Z	P-value
Shyness	0.0008	1.0009	0.0007	1.26	0.21
Activity	0.0531	1.0545	0.0831	0.64	0.52
Docility	-0.0021	0.9978	0.0084	-0.26	0.79

Table S5. Fine-gray competing risk regression model estimates for deaths attributed to predators with active foraging modes (mammalian/avian predators) and sit-and-wait, ambush predators (reptiles), with activity represented by the first component of a Principle Coordinate Analysis (PCA) of five metrics recorded in a 5-min open field test. P-values ≤0.05 are denoted with an *.

Predator	Trait	Coefficient	Exp (coefficient)	SE	Z	P-value
Active	Shyness	0.0018	1.0018	0.0008	2.10	0.04*
	Activity	0.2418	1.2735	0.1108	2.18	0.03*
	Docility	-0.0108	0.9894	0.0087	-1.25	0.21
Ambush	Shyness	0.0002	1.0001	0.0013	0.10	0.92
	Activity	-0.3063	0.7361	0.1542	-1.99	0.05*
	Docility	-0.0125	0.9876	0.0176	-0.71	0.48

Supplement 2: Testing assumptions for Cox proportional hazards regression models

We tested the assumption of proportional hazards for Cox models using the cox.zph function in the survival package (Therneau 2023). We found that distance violated the assumption of proportional hazards in the general predation model, likely because this variable had different directional relationships with ambush vs. active predators (Table S6). We reassessed this assumption for our cause-specific Cox proportional hazards models for active predators (Table S7) and ambush predators (Table S8).

Table S6: Results of test for proportional hazards for general predation model with three personality covariates, shyness (time to emerge in a latency test), activity (distance moved in the open field test), and docility (seconds frozen in a handling-bag test).

Variable	Chisq	df	p-value
Emergence	0.50	1	0.48
Activity	6.14	1	0.01
Docility	1.21	1	0.27
Global	8.48	3	0.04

Table S7: Results of test for proportional hazards for cause-specific Cox proportional hazards model for predation by active predators. The model included three personality covariates, shyness (time to emerge in a latency test), activity (distance moved in OFT), and docility (seconds frozen in a handling-bag test).

Variable	Chisq	df	p-value
Emergence	0.05	1	0.82
Activity	2.00	1	0.16
Docility	0.40	1	0.52
Global	2.77	3	0.43

Table S8: Results of test for proportional hazards for cause-specific Coz proportional hazards model for predation by ambush predators. The model included three personality covariates, shyness (time to emerge in a latency test), activity (distance moved in OFT), and docility (seconds frozen in a handling-bag test).

Variable	Chisq	df	p-value
Emergence	0.14	1	0.71
Activity	3.37	1	0.07
Docility	0.33	1	0.57
Global	4.10	3	0.25

Supplement 3: Alternative analysis of cause-specific mortalities by taxonomic groups

Although we categorized predators into two groups based on their hunting mode (active vs. ambush), it is also possible that other differences between predator groups may influence selection patterns. For this reason, we also analyzed data separately for each taxonomic group (avian, mammalian, and reptilian predators) with cause-specific Cox regressions. We observed similar directional relationships for shyness (seconds to emerge in latency tests) and activity (distance moved in the open field test) in birds and mammals (Figure S1).

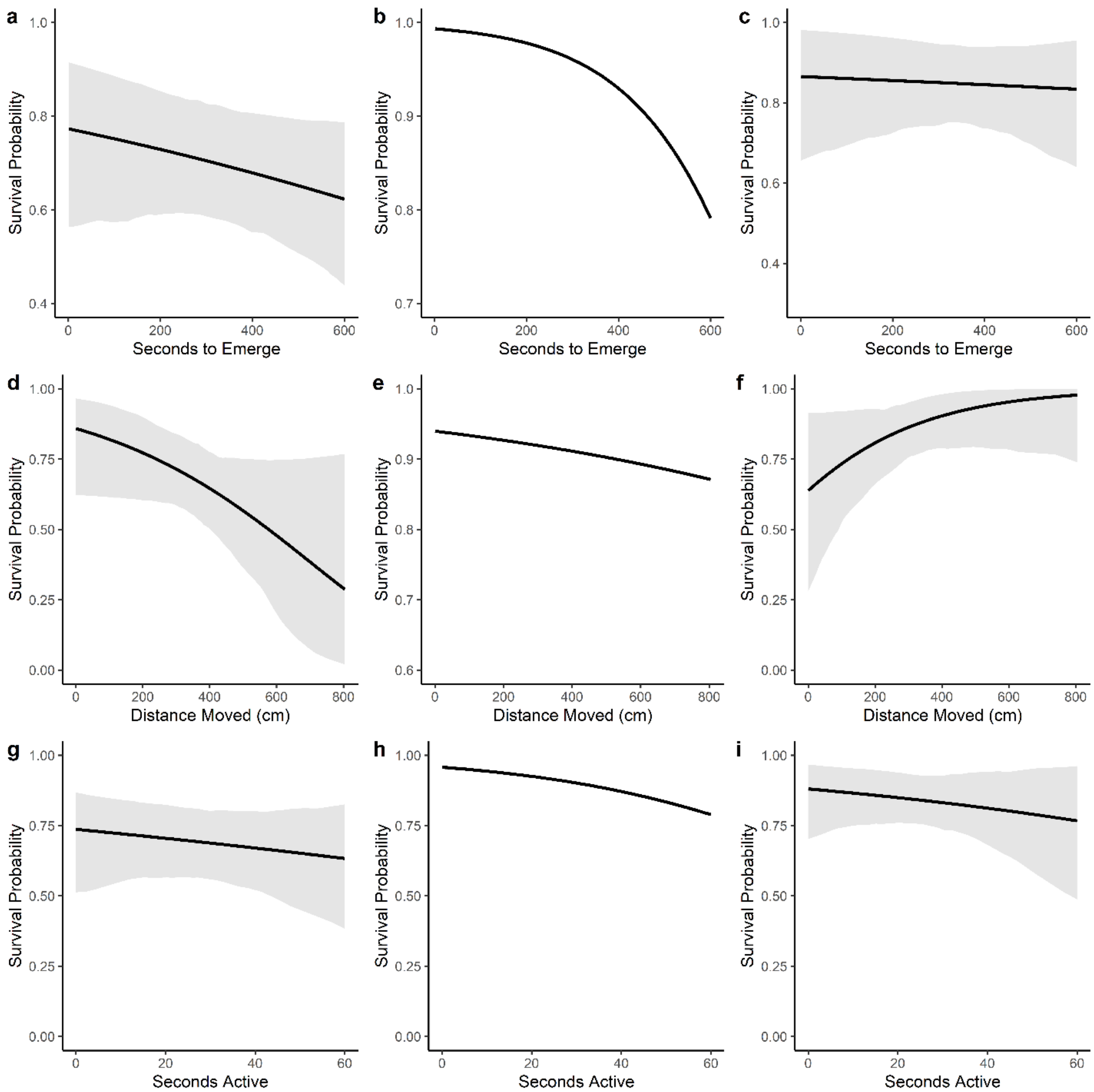

Figure S1: Relationships between personality metrics and cotton rat survival in the context of avian (column 1), mammalian (column 2), and reptilian predators (column 3). Low numbers of deaths attributed to mammalian predators (n= 6) prevented the calculation of confidence intervals.

Supplemental Materials Literature Cited

Harrell F. 2024. Hmisc: Harrell Miscellaneous. https://CRAN.R-project.org/package=Hmisc

Jolliffe IT, editor. 2002. Principal Component Analysis for Special Types of Data. In: Principal Component Analysis. Springer; p 338–372. https://doi.org/10.1007/0-387-22440-8_13

Korkmaz S, Göksülük D, Zararsiz G. 2014. MVN: An R package for assessing multivariate normality. R Journal. 6(2):151–162

Therneau TM. 2023. A package for survival analysis in R. R package version 3.5-5. https://CRAN.R-project.org/package=survival
